# Functional Precision Oncology in Fibrolamellar Carcinoma: Ex Vivo Identification of Therapeutic Vulnerabilities

**DOI:** 10.3390/cancers18111744

**Published:** 2026-05-27

**Authors:** Sabina A. Schneider, Paulo D’Amora, Steven S. Evans, Paul Kent, Tom Stockwell, Vikrant S. Bakaya, Paula J. Bernard, Federico R. Francisco, Luisa Torres, John Henry, Ismael D. C. G. Silva, Robert A. Nagourney

**Affiliations:** 1 Nagourney Cancer Institute, 750 East 29th Street, Long Beach, CA 90806, USA; saschneider5@wisc.edu (S.A.S.); pdamora@nagourneyci.com (P.D.); sevans@nagourneyci.com (S.S.E.); vbakaya@nagourneyci.com (V.S.B.); pbernard@nagourneyci.com (P.J.B.); ffrancisco@nagourneyci.com (F.R.F.); ltorres@nagourneyci.com (L.T.); jhenry@nagourneyci.com (J.H.J.); 2 Metabolomycs, Inc., 750 East 29th Street, Long Beach, CA 90806, USA; 3 Gynecology Department, School of Medicine of the Federal University of São Paulo (EPM-UNIFESP), Rua Pedro de Toledo 781, São Paulo 04039-032, SP, Brazil; dale.guerreiro@unifesp.br; 4 FibroFighters Foundation, 31805 Temecula Parkway #D7-512, Temecula, CA 92592, USA; paulkentmd@fibrofighters.org (P.K.); tom@fibrofighters.org (T.S.); 5 Department of Obstetrics and Gynecology, University of California Irvine (UC Irvine), 101 The City Drive South, Orange, CA 92868, USA

**Keywords:** fibrolamellar carcinoma, functional precision oncology, ex vivo drug sensitivity testing, programmed cell death, drug combination therapy, metabolic profile, metabolomics, personalized cancer treatment, rare liver cancer

## Abstract

**Background:** Fibrolamellar carcinoma (FLC) is a rare liver malignancy affecting adolescents. FLCs harbor a DNAJB1–PRKACA gene fusion that combines heat shock protein DNAJB1 with the catalytic subunit of protein kinase A. Surgery with systemic therapy provides 5-year survivals of 30–50%, but advanced disease remains largely incurable. Three-dimensional explants from 41 FLC patients were interrogated for drug sensitivity, resistance, and synergy against cytotoxics, targeted agents, and signal transduction inhibitors. **Methods:** Sterile specimens from histologically confirmed FLC patients were analyzed by Ex Vivo Analysis of Programmed Cell Death (EVA/PCD™) in a CLIA-licensed laboratory. Following mechanical and enzymatic disaggregation, explants underwent 72 h drug exposure. LC_50_ values were derived from five-point dose–response curves and compared with a database of over 10,000 human tumor analyses. Synergy was assessed by combination index. In parallel, targeted metabolomic profiling was performed in five FLC patients using tandem MS/MS. **Results:** Forty-one samples were analyzed. Of 24 drugs selected, tumor-cell yields were adequate for testing in 18 (75%). Single-agent activity favored vorinostat, followed by phenformin and 6-diazo-5-oxo-L-norleucine. Combinations favored gemcitabine plus oxaliplatin (GEMOX) and 5-FU plus interferon. Metabolomic analysis identified distinct signature consistent with mitochondrial dysfunction and altered polyamine metabolism. **Conclusions:** The present findings are exploratory, and hypothesis-generating and should not be interpreted as evidence of clinical efficacy. Prospective clinical validation and mechanistic studies will be required to further define the therapeutic relevance of these observations in fibrolamellar carcinoma.

## Simple Summary

Fibrolamellar carcinoma (FLC) is a rare liver cancer that primarily affects adolescents and young adults. For patients who present with advanced or recurrent disease, there are few effective treatment options. We applied a three-dimensional functional precision oncology platform, Ex Vivo Analysis of Programmed Cell Death (EVA/PCD™), to identify drug sensitivities and effective drug combinations in tumor samples from FLC patients. In parallel, we performed targeted plasma metabolomic profiling in five patients using tandem mass spectrometry to explore systemic metabolic alterations that might explain the drug-response profiles. Functional testing identified vulnerabilities to epigenetic and metabolic therapies, including vorinostat, phenformin, and 6-diazo-5-oxo-L-norleucine, while metabolomic analyses revealed distinct metabolic signatures consistent with mitochondrial dysfunction and altered polyamine metabolism. Together, these findings suggest that FLC tumors harbor actionable metabolic vulnerabilities that may offer novel therapeutic options.

## 1. Introduction

Fibrolamellar carcinoma (FLC) is a rare primary liver malignancy that is clinically and biologically distinct from hepatocellular carcinoma (HCC). FLC predominantly affects adolescents and young adults who do not present with underlying chronic liver disease, cirrhosis or viral hepatitis, in contrast to the typical demographics of HCC—found in older individuals with significant hepatopathy [1,2,3]. FLC accounts for less than 1% of all primary liver cancers, with an annual incidence in the United States of 0.02 per 100,000 [2,3]. The disease often presents insidiously with nonspecific symptoms such as abdominal pain, fatigue, and weight loss, contributing to diagnosis at more advanced stages [3,4].

Histologically, FLC is characterized by large polygonal tumor cells with abundant eosinophilic cytoplasm and prominent fibrous bands arranged in lamellae, giving rise to its eponymous designation [5]. Molecular characterization has identified a nearly ubiquitous 400 kb deletion on chromosome 19 that results in the DNAJB1–PRKACA fusion gene, which encodes for a chimeric protein that dysregulates cyclic AMP-dependent signaling pathways and drives tumorigenesis [6,7,8]. This fusion gene is highly specific for FLC and is not typically observed in HCC or other hepatic neoplasms, making it an important diagnostic and potentially therapeutic biomarker [8].

Despite younger age at presentation, the clinical course of FLC is aggressive, with high rates of recurrence, even after surgical resection, which, when combined with systemic therapy remains the only curative intervention [9]. Reported 5-year survivals vary widely but are generally in the range of 30% to 60% [9,10,11]. Recurrence-free survival is similarly poor, with recurrence rates exceeding 60% in many series [9,10]. In patients with unresectable or metastatic disease, outcomes are worse, and no universally accepted systemic therapy currently exists [1,12]. Conventional cytotoxic chemotherapies and tyrosine kinase inhibitors (TKIs) used in HCC demonstrate modest activity in FLC, reflecting intrinsic resistance and the unique biology of this tumor [12,13].

The paucity of effective systemic options has limited progress for patients with advanced or recurrent FLC. Evidence guiding therapy is primarily derived from small case series, retrospective cohorts, or extrapolation from HCC treatment paradigms rather than prospective, randomized clinical trials specific to FLC [12,14]. Molecularly guided therapeutic strategies focusing on DNAJB1–PRKACA fusion or downstream signaling have been explored in preclinical models and early translational studies, but to date, genomic profiling has not yielded broadly effective targeted therapies. This likely reflects the relatively low tumor mutational burden and lack of recurrent, targetable co-mutations beyond the defining fusion event [15,16].

Functional profiling is a precision oncology platform that assesses tumor response to drugs ex vivo, capturing dynamic phenotypic responses to therapeutic agents and combinations. Functional profiling integrates multiple aspects of tumor biology, including microenvironmental interactions and drug synergy, enabling the identification of actionable sensitivities that may not be predicted by genomic analyses. Ex vivo Analysis of Programmed Cell Death (EVA/PCD™) is a CLIA-validated functional assay technology that quantifies cell-death responses in intact tumor explants following exposure to panels of anticancer agents and rational combinations [17]. Results in a broad array of other malignancies have established the predictive validity of this platform for the selection of active drugs and combinations [17,18,19,20].

Given the urgent need for more effective therapeutic strategies in FLC, functional ex vivo drug sensitivity profiling offers an avenue to identify potentially effective treatment options in this disease. In the present study, we applied EVA/PCD™ to a multi-institutional cohort of FLC specimens to characterize drug-response profiles and identify novel therapeutic strategies for patients with FLC. In parallel, we performed targeted plasma metabolomic analysis using quantitative tandem mass spectrometry to explore systemic metabolic alterations associated with this malignancy and their potential relationship with observed therapeutic vulnerabilities.

## 2. Materials and Methods

### 2.1. Ethical and Regulatory Considerations

The functional profiling analyses described in this study were performed within a CLIA-certified laboratory environment (CLIA-05D0871981) using retrospectively analyzed, de-identified clinical and laboratory data generated during routine clinical testing and within the broader XCELSIOR precision oncology observational registry framework (ClinicalTrials.gov Identifier: NCT03793088), conducted under oversight of the Genetic Alliance IRB (IRB00003999).

Tumor specimens were submitted for clinically indicated ex vivo functional profiling as part of routine clinical care. Patients participating in the XCELSIOR registry provided informed consent permitting collection and analysis of clinical, molecular, and longitudinal outcome data within the registry framework.

In parallel, the metabolomic component of the study was conducted prospectively under WCG/WIRB-approved protocol #20162430, with written informed consent obtained from all participants prior to plasma collection and metabolomic analysis.

The functional profiling analyses presented herein represent retrospective analysis of de-identified clinical data and did not involve investigational therapeutic intervention as part of the present study.

### 2.2. Study Design and Sample Acquisition

This study was designed as a retrospective cross-sectional translational observational investigation of ex vivo functional drug sensitivity profiling in fibrolamellar carcinoma specimens submitted for clinical testing and registry-based precision oncology analysis. Reporting of the study was revised in accordance with STROBE recommendations for observational studies where applicable (Supplementary File S1). Importantly, this investigation was conducted as part of a collaborative effort between the Nagourney Cancer Institute and the FibroFighters Foundation (www.fibrofighters.org), an international patient-driven organization dedicated to advancing research and therapeutic development for this rare malignancy.

Patients with histologically confirmed fibrolamellar carcinoma (FLC) were retrospectively identified through the XCELSIOR precision oncology observational registry framework (ClinicalTrials.gov Identifier: NCT03793088). Eligible specimens included primary, recurrent, or metastatic tumor samples submitted for clinically indicated ex vivo functional profiling that yielded sufficient viable tissue for analysis.

Because of the retrospective, multicenter nature of the study and the rarity of FLC, complete clinicopathologic annotation, detailed treatment history, and molecular characterization were not uniformly available for all cases. Molecular confirmation of the DNAJB1-PRKACA fusion transcript was therefore not available for every analyzed specimen.

The majority (76.9%) of the submitted specimens were obtained from locally recurrent liver tumors. The remainder were collected from regional lymph nodes (17.3%) or cytologically positive ascitic fluid (5.8%). We did not observe significant differences in drug-response profiles between primary liver and other tumor sources, but the limited sample size makes direct comparisons difficult. Future analyses will more formally stratify according to tissue source.

Specimens were collected during clinically indicated surgical resections or image-guided biopsies at participating institutions affiliated with the FibroFighters network and were submitted fresh by overnight delivery to the Nagourney Cancer Institute. A minimum quantity of viable tumor tissue was required to ensure adequate three-dimensional explant yield for analysis. Samples were placed immediately into sterile transport media consisting of RPMI-1640 supplemented with L-glutamine, antibiotics, and heat-inactivated fetal bovine serum and shipped under controlled conditions. Tumor processing was initiated upon receipt, within 24 h of procurement.

### 2.3. Tumor Processing and Micro-Spheroid Preparation

The EVA/PCD™ platform was selected because it enables integrated phenotypic assessment of drug-induced programmed cell death within viable multicellular tumor micro-spheroids while preserving aspects of tumor architecture and microenvironmental signaling. Unlike purely genomic approaches, functional profiling allows for direct evaluation of biologically active therapeutic vulnerabilities at the phenotypic level [19,20].

This approach may be particularly relevant in rare tumors such as fibrolamellar carcinoma, where actionable genomic alterations beyond DNAJB1-PRKACA fusion are limited, and prospective randomized therapeutic trials remain difficult due to disease rarity.

Upon arrival, specimens underwent gross inspection and initial viability assessment under sterile conditions within a Class II biological safety cabinet. Tumor tissue was mechanically minced using sterile scalpels and subjected to brief enzymatic dissociation using collagenase-based protocols optimized to preserve multicellular architecture.

Following dissociation, tumor suspensions were enriched for multicellular clusters through serial centrifugation and density-based separation. The resulting preparation consisted predominantly of tumor-derived three-dimensional micro-spheroids (organoid-like clusters), preserving elements of tumor architecture, extracellular matrix, stromal components, and associated non-malignant cells. Micro-spheroids represent the functional unit of analysis within the Ex Vivo Analysis of Programmed Cell Death (EVA/PCD^®^) platform.

An aliquot of processed material was reserved for cytopathologic quality control, including hematoxylin and eosin (H&E) staining, to confirm tumor adequacy and cellular composition. Baseline viability (“Day 0”) was established prior to drug exposure.

### 2.4. Drug Selection and Exposure Conditions

Tumor micro-spheroids were exposed to a predefined panel of clinically relevant anticancer agents and rational drug combinations. The composition of this disease-informed panel was developed jointly by the FibroFighters Foundation and the Nagourney Cancer Institute to reflect contemporary clinical practice, emerging biologic insights in FLC, and investigational therapeutic strategies currently under consideration for this rare malignancy.

Accordingly, the panel incorporated cytotoxic chemotherapy backbones, targeted kinase inhibitors, epigenetic modulators, metabolic inhibitors, and apoptosis-directed agents, enabling systematic interrogation of actionable vulnerabilities within the FLC phenotype. Tested agents included retinoic acid (ATRA), alpelisib (BYL), irinotecan (CAMP), celecoxib (CCX), cobimetinib (COB), 6-diazo-5-L-norleucine (DON), everolimus (EVER), gemcitabine plus oxaliplatin (GEM + LOHP), KAT/3-bromopyruvate (KAT), lenvatinib (LVAT), navitoclax (NCLX), panobinostat (PANO), phenformin (PFN), quercetin (QUER), regorafenib (REG), vorinostat (SAHA), 5-Fluoruracil (5FU), and alpha-Interferon (INF). Agents were evaluated both as single compounds and in rational doublet or triplet combinations designed to assess potential therapeutic synergy.

Tumor suspensions were distributed into 96-well plates and exposed to drugs across clinically relevant concentration ranges. Continuous drug exposure was maintained for approximately 72 h under controlled culture conditions (37 °C, 5% CO_2_). Each condition was tested in replicate, with untreated wells serving as negative controls.

### 2.5. Assessment of Programmed Cell Death

Drug-induced cell death was quantified using morphologic and biochemical endpoints consistent with the EVA/PCD^®^ platform. Direct microscopic assessment of apoptotic and non-viable tumor cells identified cells undergoing a programmed cell-death pathway in response to drug exposure.

Quantitative analysis focused on the proportion of non-viable cells within treated micro-spheroids relative to baseline and untreated controls.

### 2.6. Dose–Response Modeling and Data Analysis

Five-point dose–response curves were generated for each agent and combination. Lethal concentration values producing 50% tumor cell death (LC_50_) were interpolated from averaged replicate data. Individual LC_50_ values were compared against population-based reference distributions derived from a large repository of prior EVA/PCD^®^ analyses across multiple solid tumor types.

Drug responses were categorized as sensitive, intermediate, or resistant based on predefined statistical thresholds relative to the reference mean and standard deviation. Drug combinations were additionally evaluated for evidence of synergistic activity when observed cytotoxic effects exceeded those predicted from individual agent activity.

All analyses were performed using proprietary analytical software developed and validated within the CLIA laboratory environment.

### 2.7. Clinical Reporting

Functional profiling results were compiled into structured clinical reports and returned to the treating oncologist. Reports summarized relative drug activity, resistance patterns, and candidate therapeutic combinations demonstrating favorable functional profiles, with the intent of informing individualized treatment selection. All therapeutic decisions remained exclusively at the discretion of the treating physician.

### 2.8. Metabolomic Analysis

Under an IRB-approved protocol, 5 FLC patients were offered the opportunity to provide plasma samples for metabolomic analysis following informed consent. Fasting blood samples were collected and processed according to standardized protocols. Plasma preparation and metabolite quantification were performed using the AbsoluteIDQ^®^ p180 kit (Biocrates Life Sciences AG, Innsbruck, Austria), a validated targeted metabolomics platform based on electrospray ionization tandem mass spectrometry (ESI-MS/MS). Briefly, plasma samples were subjected to phenyl isothiocyanate (PITC) derivatization, followed by extraction and analysis. Amino acids and biogenic amines were quantified by liquid chromatography–MS/MS (LC-MS/MS), while acylcarnitines, phospholipids, sphingomyelins, and hexoses were analyzed by flow injection analysis–MS/MS (FIA-MS/MS). Absolute quantification of 186 annotated metabolites was achieved using internal standards and calibration curves, with data acquisition performed on a SCIEX LC-MS/MS platform. Quality control was ensured through internal standards, kit-provided controls, and external reference materials.

Metabolomic data were processed using WebIDQ platform (Biocrates Life Sciences AG, Innsbruck, Austria) and exported for downstream statistical analysis in MetaboAnalyst 6.0. Log transformation and normalization were applied prior to analysis. Both unsupervised and supervised multivariate approaches were utilized, including principal component analysis (PCA) and partial least-squares discriminant analysis (PLS-DA), to identify patterns and group separation. Univariate analyses, including fold-change assessment and statistical testing, were performed to identify significantly altered metabolites. Heatmaps and clustering analyses were generated to visualize metabolic signatures, and identified metabolites were mapped to known biochemical pathways using the Human Metabolome Database (HMDB) to support biological interpretation [21].

## 3. Results

**Figure 1:**
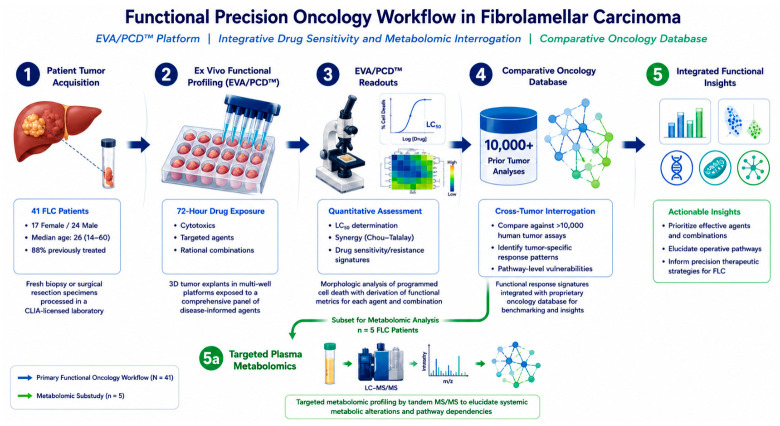
Integrative functional interrogation of therapeutic vulnerabilities in fibrolamellar carcinoma. Schematic illustration of the EVA/PCD™ functional precision oncology workflow applied to fibrolamellar carcinoma (FLC), including tumor acquisition, micro-spheroid preparation, ex vivo drug exposure, functional response analysis, and integration with metabolomic profiling. This figure was generated as an illustrative conceptual schematic using the assistance of artificial intelligence to generate the graphic and does not represent AI-generated scientific data, image analysis, statistical output, or study results. (1) Fresh tumor tissue obtained through biopsy or surgical resection is collected and transported to the laboratory for ex vivo functional profiling. (2) Tumor specimens are processed and distributed into multi-well platforms, where they are exposed to a disease-informed panel of anticancer agents, including cytotoxic chemotherapies, targeted therapies, and rational drug combinations, enabling systematic assessment of drug response. (3) Drug-induced cell death is evaluated through morphologic analysis using microscopy and specific staining approaches to identify cells undergoing programmed cell death, followed by quantitative assessment of cell-death patterns, including apoptosis, and derivation of functional metrics such as LC_50_ and drug synergy. (4) Functional response signatures are integrated with a large oncology reference database comprising over 10,000 prior analyses across multiple tumor types, allowing for comparative evaluation of drug activity and identification of tumor-specific response patterns and pathway-level dependencies. (5, 5a) In selected cases, targeted plasma metabolomic profiling using quantitative mass spectrometry (LC–MS/MS) is performed to further elucidate systemic metabolic alterations and support the identification of biologically relevant pathways, including metabolic reprogramming, epigenetic modulation, and mitochondrial targeting, thereby refining precision therapeutic strategies for this rare malignancy.

A total of 41 tumor specimens from patients with histologically confirmed fibrolamellar carcinoma (FLC) were submitted for functional ex vivo profiling through the collaborative clinical network established with the FibroFighters Foundation. The overall analytical framework of this study is summarized in Figure 1, which depicts the stepwise workflow of the EVA/PCD™ platform, including tumor acquisition, micro-spheroid preparation, systematic drug exposure, and integrative interpretation of functional response signatures within a cross-tumor oncology reference database.

Baseline demographic and clinical characteristics of the analyzed cohort are presented in Table 1. Consistent with the known epidemiology of FLC, patients were predominantly adolescents and young adults (median age of 27.5 years; range: 14–60), with a slight male predominance. Notably, the majority of specimens were obtained from patients with previously treated disease, reflecting the clinical reality of recurrent or refractory FLC at the time of tissue submission for functional interrogation.

Prior systemic therapies administered clinically before EVA/PCD™ testing are summarized in Table 2. Most patients had received multi-agent treatment regimens, frequently incorporating cytotoxic chemotherapy backbones such as gemcitabine and oxaliplatin, targeted therapies including lenvatinib, and immune checkpoint inhibition with nivolumab. These data underscore the absence of a standardized systemic treatment paradigm in FLC and highlight the need for individualized therapeutic strategies informed by functional profiling approaches.

**Figure 2:**
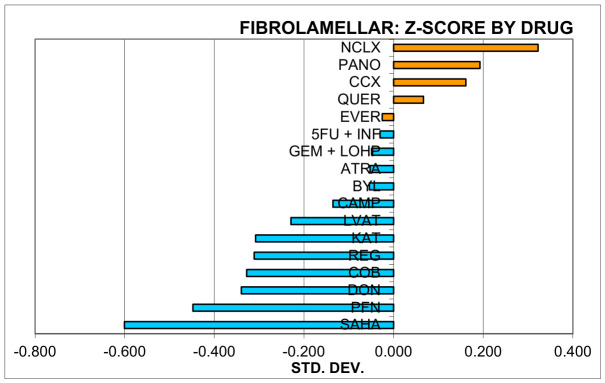
Comparative Z-score analysis of ex vivo drug sensitivity in fibrolamellar carcinoma. Horizontal bar plot showing relative drug activity in fibrolamellar carcinoma (FLC) specimens assessed by Ex Vivo Analysis of Programmed Cell Death (EVA/PCD5). For each agent or combination, LC_50_-derived functional response values obtained in FLC tumor explants were normalized against a large reference distribution of prior human solid-tumor explant analyses and expressed as Z-scores (standard deviations from the population mean). Negative Z-scores (blue bars, left) indicate increased drug-induced programmed cell death relative to the reference cohort (greater functional sensitivity), whereas positive Z-scores (orange bars, right) reflect reduced activity and relative resistance. Among tested agents, the strongest functional vulnerabilities were observed for vorinostat (SAHA; HDAC inhibition), phenformin (PFN; mitochondrial complex I inhibition), and 6-diazo-5-L-norleucine (DON; glutaminolysis inhibition), supporting a dominant metabolic–epigenetic dependency in the FLC phenotype. Notably, combination therapies also demonstrated significant activity, including gemcitabine plus oxaliplatin (GEM + LOHP) and 5-fluorouracil plus interferon (5-FU + IFN), with the latter showing enhanced sensitivity following everolimus within the response spectrum. Intermediate activity was observed for selected kinase-directed and cytotoxic regimens, whereas navitoclax (NCLX), panobinostat (PANO), celecoxib (CCX), and quercetin (QUER) demonstrated limited single-agent activity in this cohort. Abbreviations: ATRA, retinoic acid; BYL, alpelisib; CAMP, irinotecan; CCX, celecoxib; COB, cobimetinib; DON, 6-diazo-5-L-norleucine; EVER, everolimus; GEM + LOHP, gemcitabine plus oxaliplatin; KAT, KAT/3-bromopyruvate; LVAT, lenvatinib; NCLX, navitoclax; PANO, panobinostat; PFN, phenformin; QUER, quercetin; REG, regorafenib; SAHA, vorinostat; IFN, interferon.

Functional drug sensitivity patterns across the tested panel were quantitatively assessed through comparative Z-score normalization against a large reference distribution of prior human solid-tumor explant analyses. As shown in Figure 2, several agents demonstrated enhanced programmed cell-death induction in FLC specimens, as reflected by negative Z-scores indicating increased activity relative to the broader oncology cohort. The most pronounced functional vulnerabilities were observed for vorinostat (SAHA), phenformin (PFN), and 6-diazo-5-L-norleucine (DON), implicating convergent metabolic and epigenetic dependencies within the FLC phenotype. In contrast, agents such as navitoclax (NCLX), panobinostat (PANO), celecoxib (CCX), and quercetin (QUER) exhibited limited single-agent activity, emphasizing the heterogeneity of therapeutic responsiveness and the importance of rational combination strategies.

To further illustrate inter-patient variability in ex vivo functional response patterns, patient-specific SAHA (vorinostat) Z-scores relative to the overall institutional SAHA response database are provided in Supplementary Figure S1. Although heterogeneity in drug sensitivity was observed across individual FLC specimens, a subset of tumors demonstrated substantial relative sensitivity to SAHA compared with the broader institutional reference population.

### Metabolomic Profiling of Fibrolamellar Carcinoma

To further characterize the biological underpinnings of fibrolamellar carcinoma (FLC), targeted plasma metabolomic profiling was performed using quantitative tandem mass spectrometry (MS/MS) based on the Biocrates AbsoluteIDQ^®^ platform. This analysis aimed to identify systemic metabolic alterations associated with FLC and to complement the functional drug sensitivity findings by providing additional insight into underlying metabolic dependencies.

**Figure 3:**
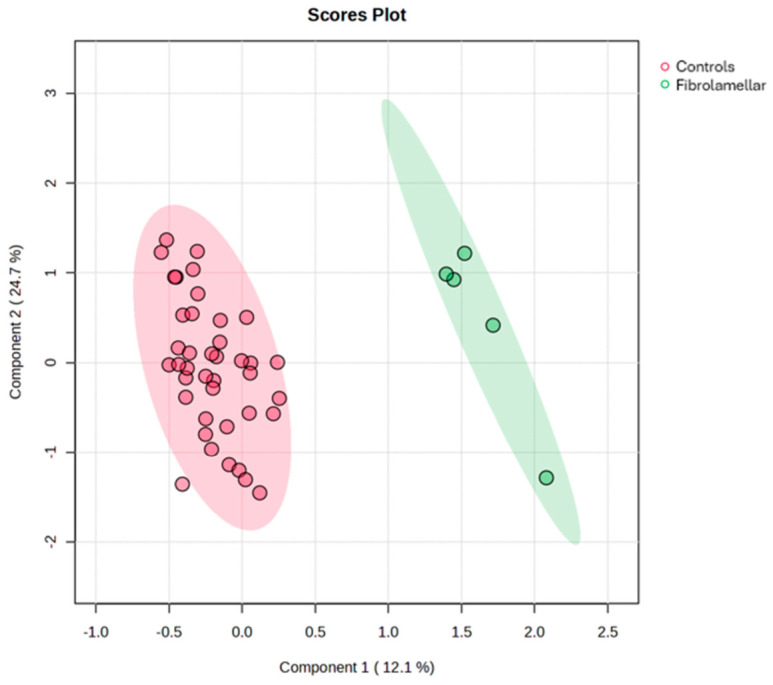
Distinct plasma metabolomic signature in fibrolamellar carcinoma identified by PLS-DA. Three-dimensional projection of metabolomic profiles comparing controls (red) and fibrolamellar carcinoma (green). Partial least-squares discriminant analysis (PLS-DA), a supervised multivariate approach, reveals clear separation between groups based on global metabolic signatures. The fibrolamellar carcinoma cohort (n = 5) was compared with age- and sex-matched controls (n = 40; median age, 60 years [range, 40–75]; 17 [43%] female and 23 [57%] male), demonstrating a distinct metabolic phenotype associated with fibrolamellar carcinoma.

Multivariate analysis was first performed to evaluate global metabolic differences between groups. As shown in Figure 3, partial least-squares discriminant analysis (PLS-DA) demonstrated a clear separation between FLC patients and matched controls, indicating the presence of a distinct metabolomic signature associated with FLC.

**Figure 4:**
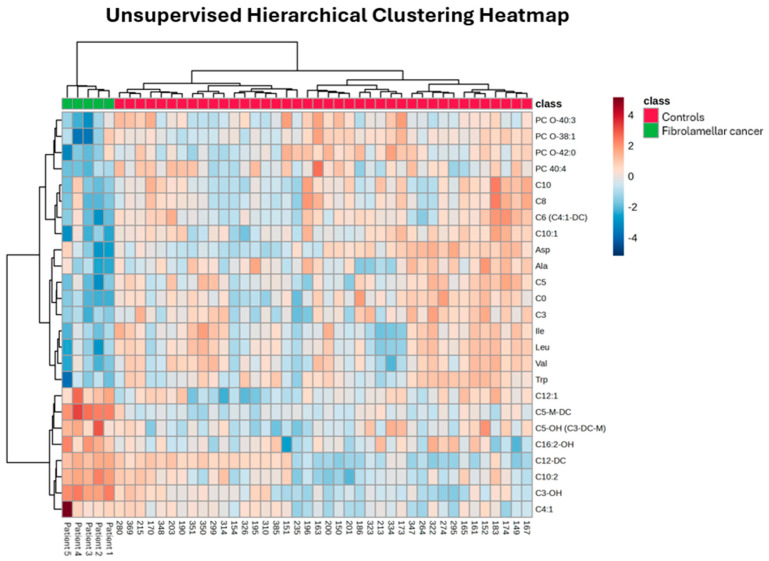
Heatmap of plasma metabolomic profiles in fibrolamellar carcinoma. Unsupervised hierarchical clustering heatmap of plasma metabolite profiles comparing fibrolamellar carcinoma (FLC) patients (green) and control subjects (red). Analysis was performed using the 25 most discriminating metabolites identified by quantitative multivariate analysis. Relative metabolite levels were normalized and scaled prior to clustering. The heatmap demonstrates distinct grouping of FLC and control samples, reflecting differences in systemic metabolic profiles. Metabolites included in the analysis are primarily related to pathways involving energy metabolism, amino acids, and polyamine metabolism.

To explore patterns of metabolite variation, unsupervised hierarchical clustering was applied to the most discriminating metabolites. As illustrated in Figure 4, heatmap analysis revealed consistent segregation of FLC and control samples, supporting the robustness of the observed metabolic differences and highlighting coordinated alterations across multiple biochemical pathways.

**Figure 5:**
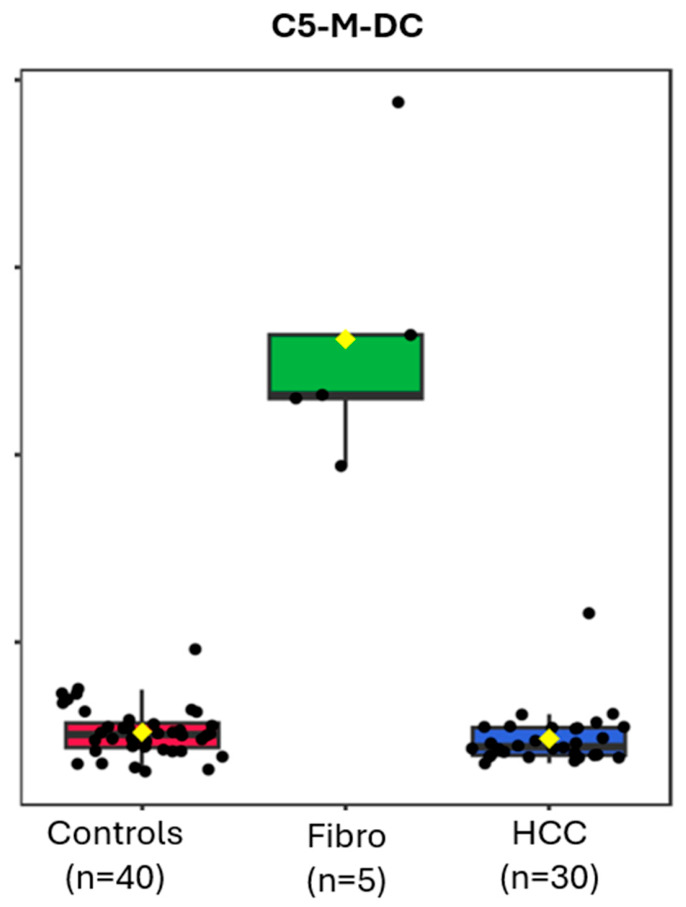
Plasma C5-M-DC concentrations across control, fibrolamellar carcinoma, and hepatocellular carcinoma groups. Box-and-whisker plot depicting plasma C5-M-DC (3-methylglutarylcarnitine) concentrations in controls (n = 40), as well as fibrolamellar carcinoma (FLC; n = 5) and hepatocellular carcinoma (HCC; n = 30) patients. Individual data points are shown for each subject. Boxes represent the interquartile range (IQR), horizontal lines indicate the median, and whiskers denote the full data range. A one-way ANOVA revealed a highly significant overall difference among groups (*p* = 8.89 × 10^−31^; FDR-adjusted *p* = 1.02 × 10^−28^). Post hoc comparisons demonstrated that C5-M-DC levels were significantly elevated in the FLC group relative to both controls and HCC, whereas controls and HCC exhibited overlapping distributions within lower concentration ranges.

To identify individual metabolites driving group discrimination, univariate analysis was performed across FLC, control, and hepatocellular carcinoma (HCC) cohorts. As shown in Figure 5, plasma levels of C5-M-DC (3-methylglutarylcarnitine) were markedly elevated in FLC compared with both controls and HCC, demonstrating strong discriminatory capacity across groups.

Collectively, these findings define a distinct metabolic phenotype associated with fibrolamellar carcinoma. The elevation of C5-M-DC, a marker of mitochondrial dysfunction, supports the presence of altered mitochondrial metabolism in FLC [22]. In parallel, reduced levels of spermine suggest disruption of polyamine metabolism, a pathway linked to tumor progression and MYC-regulated metabolic programs [23].

Taken together, these metabolomic alterations are concordant with the functional drug sensitivity results and reinforce a model in which mitochondrial dysfunction and metabolic reprogramming represent key biological features and potential therapeutic vulnerabilities in fibrolamellar carcinoma.

## 4. Discussion

The rarity of FLC and its unique genomic features have rendered this rare malignancy that afflicts children and young adults a therapeutic challenge. Conventional chemotherapies like Gemcitabine, Oxaliplatin and 5-FU have been applied with limited success. The advent of targeted therapies like the multi-targeted tyrosine kinase inhibitor lenvatinib, when combined with cytotoxic drug combinations like gemcitabine plus oxaliplatin (Gem/Ox), have provided improved responses, leading to the application of this drug triplet in the neoadjuvant setting [24]. Despite some advances, FLC in its later stages remains a highly drug-refractory and largely incurable disease.

Human tumor primary culture analyses for the selection of drugs and combinations have gained attention as new technologies allow investigators to interrogate individual patient tumors to explore novel agents and combinations [25,26,27].

We have applied human tumor analyses in a broad array of malignancies and updated results from over 10,000 individual patient studies that revealed improved response (*p* < 0.001) and one-year survival (*p* = 0.02), as we reported [28].

The principle applied in the current study borrows from our prior work that defined the utility of human tissue analyses, stipulating that ex vivo analyses correlate with clinical response if (1) the mechanisms of action in vitro are related and proportional to the mechanism of action in vivo or (2) the mechanisms of resistance in vitro are related and proportional to the mechanism of resistance in vivo [29].

We further defined chemotherapeutic drugs as probes of human biology whereby drugs with well-characterized mechanisms of action can be used to elucidate features of tumor biology [30].

The results of this study identify the highest activity for agents associated with cellular metabolism. The first, vorinostat, is a pan-HDAC inhibitor that serves as an epigenetic regulator. We previously reported that vorinostat activity strongly correlates with JQ1, a bromodomain inhibitor and tool compound for the interrogation of MYC upregulation [31]. We also tested panobinostat, a potent pan-HDAC inhibitor closely related to vorinostat. The discrepancy between these mechanistically related agents reflects an inadvertent artifact that was introduced by our inclusion of a majority of FLC patients (64%) in the panobinostat dataset. Furthermore, panobinostat activity was compared with a drug-sensitive population of multiple myeloma patients, as multiple myeloma is the FDA-approved indication for this drug. To the contrary, FLC patients in the vorinostat dataset were compared with a large number of drug-sensitive and drug-resistant tumor types, with FLC constituting only 11% of the vorinostat dataset. We believe that the vorinostat data is more reflective of FLC biology.

The second agent with metabolic activity is phenformin, a biguanide that inhibits mitochondrial metabolism at the level of Complex I [32,33]. The third agent, DON, is a glutamine inhibitor that deprives metabolically active cells of this essential amino acid. This MYC-regulated metabolic pathway is an emerging target for cancer therapy [34,35].

The findings suggest that FLC, under the influence of DNAjb1-PRKAKA, undergoes metabolic re-programming consistent with the upregulation of the protooncogene MYC. This has the capacity to drive nutrient pathways, downregulate immune response and enhance cellular survival. Using small molecule inhibitors of HDAC, glutamine metabolism and mitochondrial complex I, we recognized features of metabolic alterations and undertook a pilot analysis on 5 of the 41 FLC patients.

This pilot study used targeted mass spectrometry to examine altered metabolism as a potential driver of FLC carcinogenesis and disease progression. Among the findings was an increase in the plasma concentration of 3-methylglutarylcarnitine (C5-M-DC). This metabolite is associated with altered leucine catabolism and changes in mitochondrial flux involving HMG-CoA and mitochondrial β-oxidation. The finding of an altered mitochondrial metabolism as a driver of FLC may, in part, explain the aggressiveness of this tumor and its relative resistance to conventional chemotherapies.

There are many shortcomings of this study that must be incorporated into our discussion. The first is that this study was conducted upon previously treated patients. Prior exposure to drugs, targeted agents and immune therapies may have influenced many of the findings. As cancer represents an evolutionary process, the stressors introduced by prior therapy may have induced the observed drug-response profiles and metabolic alterations. Future studies will focus on accruing more newly diagnosed patients to allow for a better assessment of treatment effect. The second is the heterogeneous nature of the patient population by age, treatment history, sex and other potentially confounding co-variables. Future studies may allow us to better control the degree of heterogeneity or allow for stratification.

A further weakness reflects the variable tumor yield between submitted specimens. As a result, it was not possible to test all of the therapeutic agents and combinations. In instances of limited viable explant availability, testing prioritization followed predefined disease-informed therapeutic panel selection. This may have introduced some bias, as sample yield and quality could introduce artifacts into the dose-response curves, as underlying tumor robustness (drug resistance) could influence LC50 results. Future analyses including larger and more viable tissue samples should help control this influence upon the results and focus on technical feasibility considerations rather than observed response patterns.

We acknowledge that retrospective rare-tumor studies may inherently introduce referral and selection biases, which should be considered when interpreting these findings.

Importantly, ex vivo functional drug sensitivity does not necessarily predict in vivo therapeutic efficacy. In vitro observations must be viewed as surrogate markers for therapeutic response. Clinical response may be influenced by factors not fully recapitulated within ex vivo systems, including pharmacokinetics, systemic metabolism, host immune interactions, tumor evolution, and additional microenvironmental influences.

Similarly, many submitted tumor specimens originated from previously treated patients, and prior therapies may have influenced both tumor biology and observed functional drug sensitivity profiles.

An additional limitation of the present study is the extremely small sample size of the metabolomic cohort (n = 5). Given the rarity of fibrolamellar carcinoma and the limited availability of prospectively collected plasma samples, the metabolomic findings should be considered highly preliminary and hypothesis-generating rather than definitive biomarker conclusions.

The observed metabolomic signatures demonstrated biologic concordance with the ex vivo sensitivity patterns identified for phenformin and DON. Specifically, elevations in metabolites associated with mitochondrial dysfunction, altered branched-chain amino acid metabolism, and impaired oxidative metabolic flux support the hypothesis that fibrolamellar carcinoma may exhibit dependence on mitochondrial oxidative phosphorylation and glutamine-associated metabolic pathways.

Phenformin inhibits mitochondrial complex I and oxidative phosphorylation, whereas DON interferes with glutamine utilization and glutaminolysis. Together, these findings support the hypothesis that mitochondrial and glutamine-associated metabolic reprogramming may represent biologically relevant vulnerabilities in FLC.

The marked elevation of C5-M-DC (3-methylglutarylcarnitine) is particularly intriguing, as this metabolite is associated with leucine degradation, ketogenesis, and mitochondrial metabolic flux. Elevated circulating levels of C5-M-DC have been linked to impaired mitochondrial oxidative metabolism and altered branched-chain amino acid utilization and may reflect dysregulation of pathways involving HMG-CoA lyase (HMGCL), AUH, and related mitochondrial enzymes.

Given the rarity of fibrolamellar carcinoma and the exploratory nature of the present investigation, the findings reported herein should be considered preliminary and hypothesis-generating. Although biologically relevant therapeutic vulnerabilities were identified through functional and metabolomic analyses, prospective validation studies integrating clinical outcomes, molecular profiling, and mechanistic investigations will be required to provide support for future clinical implementation.

Although longitudinal clinical outcome tracking is supported within the XCELSIOR observational registry framework, formal correlation analyses between ex vivo functional profiling results and real-world patient outcomes were not systematically performed in the present study because complete longitudinal follow-up data were not uniformly available for all analyzed retrospective FLC cases.

Future prospective integration of ex vivo functional profiling with longitudinal clinical outcome data, histopathologic treatment-response assessment, and molecular characterization will be important for determining the predictive clinical utility of this approach.

## 5. Conclusions

FLC is a rare and potentially lethal malignancy that almost exclusively afflicts young people. Despite some improvements, the disease has largely proven refractory to conventional therapies. Through the dedicated efforts of the FibroFighters Foundation, this pilot study provides provocative insights into the biology of this rare disease. The results suggest that DNAjb1-PRKAKA gene re-arrangement drives metabolic re-programming consistent with upregulation of proto-oncogene MYC. This appears to confer resistance to many conventional drugs but may offer new avenues for therapy that could include epi-genetic targets and metabolic pathways. The growing recognition of the role of altered metabolism in cancer may allow for the development of new classes of drugs capable of downregulating cancer-associated metabolic re-programming with the hope of providing new therapeutic strategies for FLC patients.

## Figures and Tables

**Table 1:** Baseline demographic characteristics of patients with fibrolamellar carcinoma.

Characteristic	Value n (%)
Total patients	41 (100)
Female	17 (41)
Male	24 (59)
Mean age (years)	29.6
Median age (years)	27.5
Age range (years)	14–60
Previously treated	36
Untreated	5

Baseline demographic and treatment status of patients with histologically confirmed fibrolamellar carcinoma (FLC) whose tumor specimens were submitted for functional ex vivo profiling.

**Table 2:** Prior systemic therapies administered clinically in patients with fibrolamellar carcinoma.

Agent	Count	Percentage (%)
Gemcitabine (GEM)	26	72
Lenvatinib (LVAT)	26	72
Oxaliplatin (LOHP)	22	61
Nivolumab (NIVO)	21	58
5-Fluorouracil (5FU)	11	31
Interferon (INF)	11	31
Cisplatin (CDDP)	4	11
Carboplatin (CARBO)	3	8
Capecitabine (XELODA)	3	8
Quercetin (QUER)	3	8
Doxorubicin (DOX)	2	6
Sorafenib (NEX)	2	6
Everolimus (EVER)	2	6
Celecoxib (CCX)	2	6
Irinotecan (CAMP)	1	3
Vincristine (VCR)	1	3
Paclitaxel (TAX)	1	3
Dexamethasone (DEX)	1	3
Bevacizumab (BEV)	1	3
Retinoic Acid (ATRA)	1	3

Prior systemic therapies administered clinically before tumor submission for EVA/PCD™ testing. Percentages reflect the proportion of patients who received each agent during their clinical course; patients may have received multiple regimens.

## Data Availability

Data utilized in the preparation of this manuscript is reported in the manuscript.

## Supplementary Materials

The following supporting information can be downloaded at: https://www.mdpi.com/article/10.3390/cancers18111744/s1, Figure S1: Patient-specific SAHA (vorinostat) activity in fibrolamellar carcinoma relative to the overall institutional SAHA response database; File S1: Completed STROBE checklist.
