# Supplementary material for: Functional Precision Oncology in Fibrolamellar Carcinoma: Ex Vivo Identification of Therapeutic Vulnerabilities
Source: Cancers (Basel). 2026 May 27;18(11):1744. doi: 10.3390/cancers18111744 (PMC13255816; doi:10.3390/cancers18111744)
Supplement: Supplementary file 1 [file cancers-18-01744-s001.zip › cancers-4285227-supplementary-File S1-Nagourney_STROBE_Assessment.pdf]

STROBE Checklist Assessment and Recommendations for the Nagourney FLC Manuscript

This document provides an item-by-item assessment of the STROBE (Strengthening the Reporting of Observational Studies in Epidemiology) checklist as applied to the manuscript 'Functional Precision Oncology in Fibrolamellar Carcinoma: Ex Vivo Identification of Actionable Therapies.' Each item includes an assessment of current manuscript compliance and specific recommendations for improvement.

1(a) Study design in title/abstract

Current Status: Addressed.

Recommendation: Add terminology such as 'retrospective observational study' or 'retrospective cohort study' to the title or abstract.

1(b) Informative abstract

Current Status: Addressed.

Recommendation: Add a brief limitation statement noting that findings are hypothesis-generating and require prospective validation.

2. Background/rationale

Current Status: Well addressed.

Recommendation: No major revisions required.

3. Objectives

Current Status: Adequately addressed.

Recommendation: Explicitly state prespecified hypotheses regarding metabolic and epigenetic vulnerabilities in FLC.

4. Study design

Current Status: Adequately addressed.

Recommendation: Move the retrospective study-design description earlier in the manuscript for clarity.

5. Setting

Current Status: Addressed.

Recommendation: Add dates of recruitment, specimen collection, and study period.

6(a). Participants

Current Status: Addressed.

Recommendation: Explicitly define inclusion/exclusion criteria, prior therapy status, and eligibility requirements.

6(b). Matching criteria

Current Status: Addressed.

Recommendation: State explicitly that no matching procedures were used.

7. Variables

Current Status: Addressed.

Recommendation: Clearly define primary and secondary outcomes, synergy criteria, and definitions of sensitivity/resistance.

8. Data sources/measurement

Current Status: Well addressed.

Recommendation: No major revisions required.

9. Bias

Current Status: Addressed.

Recommendation: Discuss referral bias, selection bias, survivorship bias, and assay interpretation bias.

10. Study size

Current Status: Addressed.

Recommendation: Explain that all available eligible FLC specimens during the study period were included due to disease rarity.

11. Quantitative variables

Current Status: Addressed.

Recommendation: Clarify normalization methods, thresholds for activity, and handling of continuous variables.

12(a). Statistical methods

Current Status: Addressed.

Recommendation: Specify statistical tests, software, confidence intervals, significance thresholds, and correction methods.

12(b). Subgroups/interactions

Current Status: Addressed within the limitations of the small sample size.

Recommendation: State whether subgroup analyses were prespecified or not performed due to sample size.

12(c). Missing data

Current Status: Addressed.

Recommendation: Describe how missing or nonevaluable samples were handled.

12(d). Loss to follow-up

Current Status: Not applicable.

Recommendation: State that the study was not longitudinal and did not include follow-up analyses.

12(e). Sensitivity analyses

Current Status: Addressed. Performance characteristics of platform provided.

Recommendation: State whether sensitivity analyses were performed.

13(a). Participant flow

Current Status: Addressed.

Recommendation: Add a flow diagram showing specimen acquisition, exclusions, evaluable samples, and analyses.

13(b). Non-participation

Current Status: Addressed in the explanation of tissue procurement and patient flow.

Recommendation: Explain reasons for exclusion or non-evaluable specimens.

13(c). Flow diagram

Current Status: Provided.

Recommendation: Strongly recommend inclusion of a specimen flow diagram.

14(a). Descriptive data

Current Status: Addressed.

Recommendation: Add a baseline demographics table including age, sex, disease stage, and prior therapies.

14(b). Missing data

Current Status: Addressed.

Recommendation: Report missing data for all major variables.

14(c). Follow-up time

Current Status: Not applicable.

Recommendation: No longitudinal follow-up was performed.

15. Outcome data

Current Status: Adequately addressed.

Recommendation: Consider adding more quantitative summaries and tables.

16(a). Main results

Current Status: Addressed.

Recommendation: Include effect sizes, comparative metrics, and confidence intervals where feasible.

16(b). Category boundaries

Current Status: Not applicable.

Recommendation: Clarify if continuous variables were categorized.

16(c). Absolute risk

Current Status: Not applicable.

Recommendation: No clinical risk model was evaluated.

17. Other analyses

Current Status: Addressed.

Recommendation: Further detail metabolomic and synergy analyses.

18. Key results

Current Status: Well addressed.

Recommendation: No major revisions required.

19. Limitations

Current Status: Addressed.

Recommendation: Add a dedicated limitations paragraph discussing retrospective design, sample size, selection bias, lack of clinical outcome validation, and assay limitations.

20. Interpretation

Current Status: Addressed.

Recommendation: Further temper conclusions by emphasizing exploratory and hypothesis-generating nature.

21. Generalisability

Current Status: Addressed.

Recommendation: Discuss applicability to broader FLC populations and limitations due to rare disease sampling.

22. Funding

Current Status: Fully addressed.

Recommendation: Funding, consent, IRB status, and conflicts are appropriately disclosed.

Overall Assessment: The manuscript substantially addresses many major STROBE domains, particularly rationale, assay methodology, and biological interpretation. The most important areas requiring improvement are explicit reporting of limitations, participant selection criteria, statistical methods, handling of missing data, and inclusion of descriptive patient characteristics and specimen flow information.
