# Supplementary figures and images for: Functional Precision Oncology in Fibrolamellar Carcinoma: Ex Vivo Identification of Therapeutic Vulnerabilities
Source: Cancers (Basel). 2026 May 27;18(11):1744. doi: 10.3390/cancers18111744 (PMC13255816; doi:10.3390/cancers18111744)

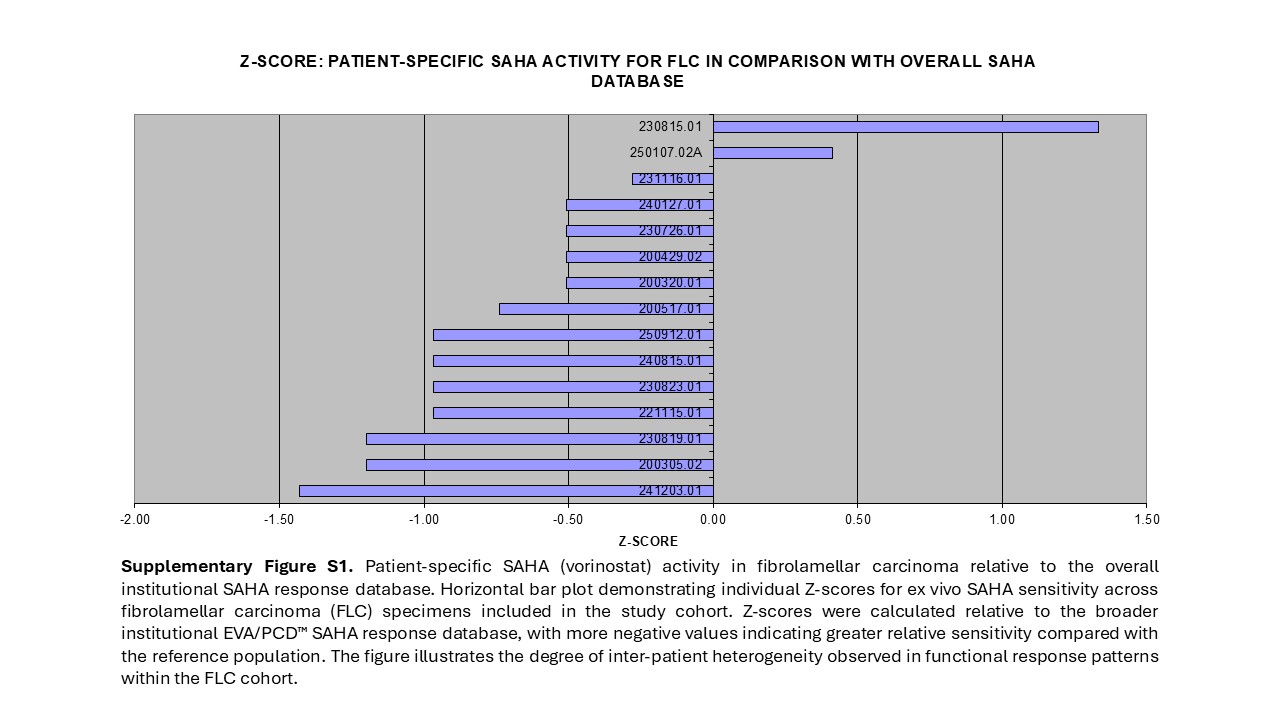

Supplement: Supplementary file 1 [file cancers-18-01744-s001.zip › cancers-4285227-supplementary Figure S1.jpg]
